# Heterojunction-Engineered g-C_3_N_4_/TiO_2_ Nanocomposites with Superior Bilirubin Removal Efficiency for Enhanced Hemoperfusion Therapy

**DOI:** 10.3390/molecules30132729

**Published:** 2025-06-25

**Authors:** Lingdong Meng, Shouxuan Tao, Liyao Wang, Yu Cao, Jianhua Hou, Chengyin Wang

**Affiliations:** 1 Hemodialysis Center of Yangzhou Hospital of Traditional Chinese Medicine, Yangzhou 225000, China; mengling-dong1982@163.com; 2 College of Chemistry and Chemical Engineering, Yangzhou University, Yangzhou 225000, China; shouxuantao@foxmail.com; 3 Faculty of Materials Science and Chemistry, China University of Geosciences, Wuhan 430074, China; WangLiYao@cug.edu.cn; 4 College of Environmental Science and Engineering, Yangzhou University, Yangzhou 225000, China

**Keywords:** g-C_3_N_4_/TiO_2_, bilirubin (BR), hemoperfusion, adsorbent

## Abstract

The g-C_3_N_4_/TiO_2_ intercalation composite material was successfully synthesized and used as the adsorbent in the hemoperfusion device. Then, the cytotoxicity and hemolysis rate were studied. The experimental results proved that g-C_3_N_4_/TiO_2_ was non-toxic to cells and would not cause hemolysis. The adsorption and removal performance of the composite material for bilirubin (BR) was explored as well. The maximum adsorption capacity for BR was 850 mg·g^−1^. Compared with the chemical hemoperfusion adsorbent coconut shell activated carbon (AC), the g-C_3_N_4_/TiO_2_ material presented excellent adsorption performance. Furthermore, SEM, infrared spectroscopy, XPS and other characterizations results indicated that g-C_3_N_4_/TiO_2_ has an effective adsorption effect on bilirubin, and the main adsorption mechanism is chemical adsorption. This study demonstrates that g-C_3_N_4_/TiO_2_ may be a potential adsorbent for hemoperfusion in the treatment of hyperbilirubinemia.

## 1. Introduction

Bilirubin (BR) is not only the main product of hemoglobin metabolism, but also the key metabolite of iron porphyrin compounds in organisms [1,2,3]. It is one of the focal pigments in human bile and an endotoxin with high toxicity [4,5,6,7]. Under normal circumstances, BR is transported to the liver in combination with albumin, then metabolized by the liver [8]. BR in healthy people is generally 1–10 mg·L^−1^ [9], but the body of patients with liver failure cannot remove BR in time, resulting in the continuous accumulation of BR in the blood. Excessive accumulation will cause harm and become hyperbilirubinemia [10]. Additionally, excessive BR accumulation may cause irreversible harm to the brain, tissues, organs and nervous system, and may even lead to death [11]. Thus, timely reduction of excessive BR concentration in a patient’s blood is very important for patients with hepatic failure, which is also a huge clinical challenge. Hemodialysis is a commonly used blood purification therapy in the market, but it cannot effectively remove BR, because BR belongs to a protein-binding toxoid. There will be specific binding between the BR toxin and albumin, the volume will become larger after binding, and it will become more difficult for BR to pass through the dialysis membrane [12].

The combination of hemoperfusion [13,14,15,16,17] and hemodialysis [18,19,20,21] is a clinically efficient and feasible method for the removal of BR toxins in organisms. Conventional hemodialysis can only remove some small molecule toxins, and the removal effect of endotoxins of large size is not ideal. The side combined with hemoperfusion can significantly enhance the removal of medium molecule and macromolecular toxins. With the development of new adsorbent materials [22,23,24,25] and the continuous improvement of adsorbent microencapsulation technology [26,27,28,29], hemoperfusion technology has made wider application progress in clinical practice [30,31,32,33]. Although hemoperfusion can effectively remove macromolecular toxins from patients and alleviate patients’ complications, there are still patients with low quality of life, high anti-disease rate and high mortality after treatment. In order to improve this situation, patients need blood purification treatment many times, and the treatment cycle becomes longer, which increases the physical and economic burden on patients. At present, the adsorbent used to remove BR in-clinic is mainly activated carbon (AC) [34,35,36], but the binding capacity of AC is weak, and the exchange resin’s specific surface area is low. These limiting factors lead to unsatisfactory treatment effect. In recent years, researchers have developed various advanced porous materials, for instance metal organic frameworks (MOFs), which are used to remove the protein-binding toxin BR in the blood of organisms [37,38,39,40]. However, the stability of a metal organic framework in liquid solution is poor, which limits its further application in blood purification. Therefore, considering the weak points and shortcomings of existing adsorbing materials, it is important to develop highly efficient adsorbents with high binding capacity and excellent mechanical stability to effectively remove BR accumulated in organisms.

Usually, toxin adsorption is based on carbon-based materials [41,42]. For instance, microporous active carbon spheres, single- or multi-wall carbon nanotubes [43,44] and three-dimensional (3D) porous graphene [45] have microporous and mesoporous structures. As a photocatalytic adsorbent, titanium dioxide (TiO_2_) can not only break down BR under ultraviolet (UV) light, it also adsorbs BR molecules [46]. Yang et al. established a detection method for BR by preparing TiO_2_ film and quartz microcrystalline balance technology [47]. The surface morphology and functional groups of TiO_2_ were controlled by different treatment temperature and time during preparation, thus improving the adsorption of BR [48,49].

Herein, we combine graphite carbon nitride (g-C_3_N_4_) with anatase TiO_2_ nanoparticles to prepare intercalated composites with high adsorption performance, high mechanical stability and excellent biocompatibility. As a blood perfusion material, it is applied to the research of blood purification. It is a potential adsorbent material for blood perfusion, and is expected to have excellent potential in the field of biological blood purification.

## 2. Results and Discussion

**Figure 1:**
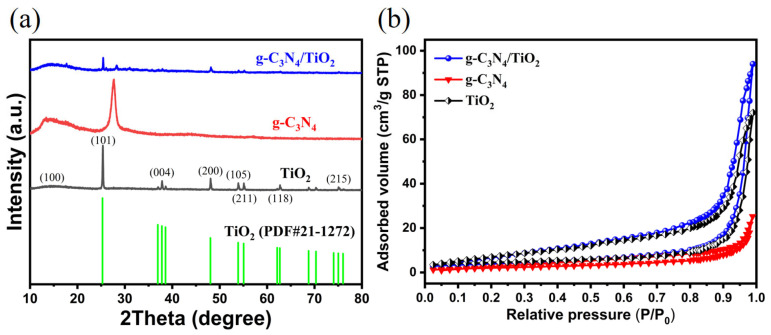
(**a**) XRD patterns of TiO_2_, g-C_3_N_4_ and g-C_3_N_4_/TiO_2_ composite materials. (**b**) N_2_ adsorption–desorption isotherms of TiO_2_, g-C_3_N_4_ and g-C_3_N_4_/TiO_2_ composites.

The composition and crystal structure of the as-prepared materials were characterized by X-ray diffraction (XRD) patterns. As shown in Figure 1a, the diffraction peak of g-C_3_N_4_ at 13.2° corresponds to the (100) crystal plane, and a distinct diffraction peak near 27.5° indicates the (002) crystal plane. The XRD pattern of TiO_2_ exhibits characteristic peaks typical of the anatase phase (JCPDS card No. 21-1272). In the g-C_3_N_4_/TiO_2_ composite, diffraction peaks corresponding to both g-C_3_N_4_ and anatase TiO_2_ are simultaneously detected. However, the lower peak position observed for the (001) plane may be attributed to the disruption of the carbon nitride layers caused by the intercalation of titanium dioxide nanoparticles. All the above results demonstrate the successful synthesis of the g-C_3_N_4_/TiO_2_ intercalated composite material.

To study the specific surface area and pore structure parameters of the material, N_2_ adsorption–desorption isotherm experiments were employed (shown in Figure 1b and Table S1). It is clear that g-C_3_N_4_ has a small BET area (7.68 m^2^·g^−1^). This is due to the tightly stacked layers. The extensive mutual contact between surfaces results in the low BET of bulk g-C_3_N_4_. The BET surface of the original TiO_2_ is 24.37 m^2^·g^−1^. Then, as combined with the intercalation of TiO_2_ nanoparticles, the specific surface area of the g-C_3_N_4_/TiO_2_ composite material increased to 34.52 m^2^·g^−1^, which is beneficial for the adsorption process due to the enhanced adsorption capacity and the increased number of adsorption sites.

**Figure 2:**
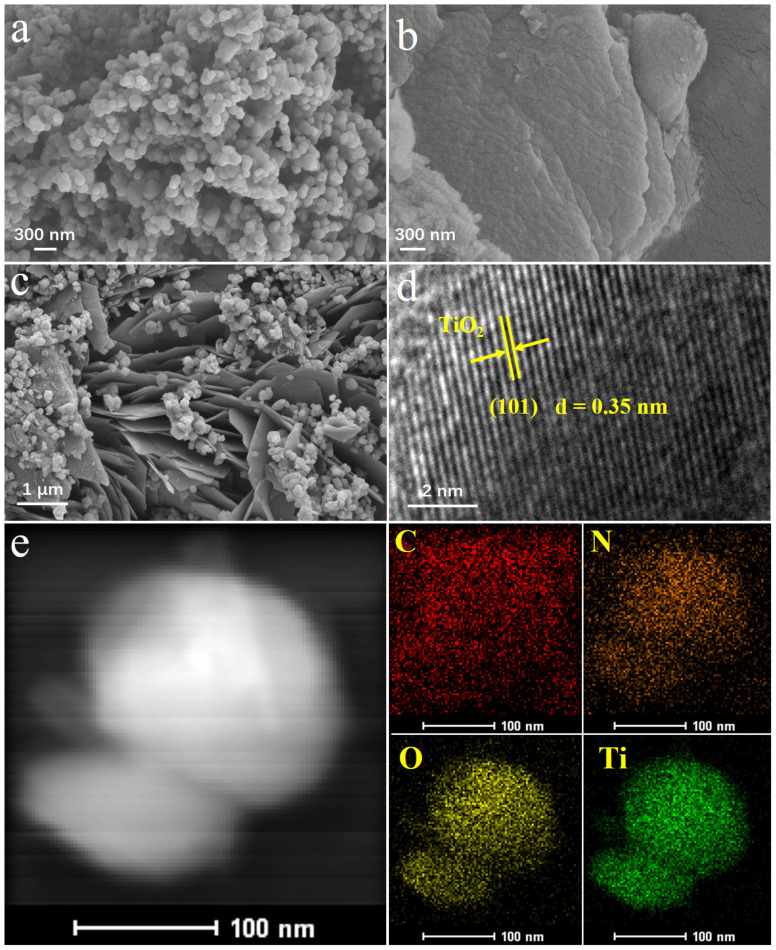
SEM images of (**a**) anatase TiO_2_, (**b**) g-C_3_N_4_, (**c**) g-C_3_N_4_/TiO_2_, (**d**) HRTEM image of g-C_3_N_4_/TiO_2_ and (**e**) the TEM-EDX of g-C_3_N_4_/TiO_2_.

The microstructure and morphology of anatase TiO_2_, g-C_3_N_4_, and the prepared g-C_3_N_4_/TiO_2_ composites were observed with a scanning electron microscope (SEM) and a transmission electron microscope (TEM). As shown in Figure 2a, anatase TiO_2_ displays smooth spherical particles with sizes of about 100 nm. A typical SEM image of g-C_3_N_4_ is shown in Figure 2b. It can be seen that pure g-C_3_N_4_ possesses an irregular fish scale stacking structure, with each layer having a thickness of approximately 50 nm. However, the tight stacking between layers limits the contact area between the material and the target species. This severely limits the adsorption capacity of bulk g-C_3_N_4_. Figure 2c shows the morphology of the prepared g-C_3_N_4_/TiO_2_ intercalated composite material. The extended g-C_3_N_4_ layers enabled TiO_2_ to disperse in spherical form within the interlayers; the g-C_3_N_4_ “baffles” effectively dispersed the clustered TiO_2_ particles. This can increase the contact area between the composite material and the target adsorbent. Figure 2d is the HRTEM image of g-C_3_N_4_/TiO_2_. The good crystalline characteristics of the material can be observed, and the corresponding lattice spacing of 0.35 nm can be indexed to the (101) plane of TiO_2_ in the g-C_3_N_4_/TiO_2_ intercalated composite material.

To further investigate the composition of g-C_3_N_4_/TiO_2_, the elemental distribution of the g-C_3_N_4_/TiO_2_ intercalated composite material was analyzed using TEM-EDX. As clearly revealed in Figure 2e, the sample primarily contains four elements: C, N, O and Ti. Moreover, the elemental mapping reveals that C, N, O and Ti are distributed very homogeneously. This further demonstrates the successful preparation of the g-C_3_N_4_/TiO_2_ composite material.

**Figure 3:**
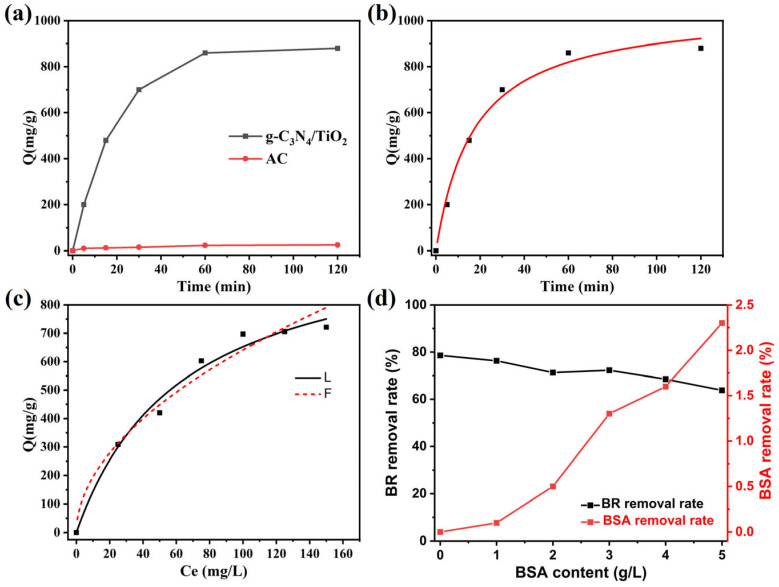
(**a**) The adsorption capacity of g-C_3_N_4_/TiO_2_ composite material and AC to BR changes with time. (**b**) The effect of adsorption time on the adsorption of BR by the g-C_3_N_4_/TiO_2_ composite. (**c**) Adsorption isotherm of g-C_3_N_4_/TiO_2_ composite material to BR aqueous solution at 37 °C. The experimental data was fitted with Freundlich (dashed line) and Langmuir (solid line) adsorption isotherm equations. (**d**) Removal efficiency of g-C_3_N_4_/TiO_2_ intercalation composites for BR and BSA in the presence of different concentrations of BSA (the BR concentration is 150 mg·L^−1^).

To study the adsorption rate of BR in blood on g-C_3_N_4_/TiO_2_ intercalation composite materials, the effect of the adsorption time on adsorption capacity was explored in the experimental design. Figure 3a displays the effect of different adsorption time on the adsorption capacity of g-C_3_N_4_/TiO_2_ intercalation composite for BR in human blood. It is obvious that the adsorption rate of BR on g-C_3_N_4_/TiO_2_ intercalation composite is the fastest in the first 20 min, and more than 90% of BR is cleared. The adsorption state reached equilibrium after approximately 60 min, and the solution density did not change over time. The adsorption capacity of g-C_3_N_4_/TiO_2_ intercalated composite for BR reaches an amazing 850 mg∙g^−1^, while the adsorption capacity of AC for BR is only 25 mg∙g^−1^, and the adsorption capacity of g-C_3_N_4_/TiO_2_ intercalated composite is 34 times that of activated carbon. This shows that the prepared g-C_3_N_4_/TiO_2_ intercalation composite has higher adsorption performance, faster adsorption rate and higher removal rate than commercial carbon nitride, which means that it can significantly reduce the cumulative concentration of BR in patients’ body and decrease the physical damage of toxins to patients, so as to alleviate the condition of patients faster.

The fitted image is shown in Figure 3b, and the K_2_ value calculated is 0.0093 g/(mg·h). K_2_ is a rate constant, and its lower value shows that the adsorption rate decreases over time, as well as that the adsorption rate will increase with the quantity of non-adsorption sites. The more sites, the faster the adsorption. After fitting with the quasi second-order kinetic equation, the correlation coefficient R^2^ (R^2^ = 0.988) of the linear graph is very close to 1, which indicates that the adsorption process is more in line with the quasi second-order kinetic equation. Quasi second-order kinetic adsorption shows that the adsorption process is a kind of chemical adsorption, and chemical adsorption refers to the chemical reaction between BR and g-C_3_N_4_/TiO_2_ intercalation composite; this implies that the process of adsorption will have the formation of chemical bonds. Therefore, this may show that some elements on the superficies of g-C_3_N_4_/TiO_2_ intercalation composite can form chemical bonds with some elements in BR, resulting in chemical adsorption.

Figure 3c shows the adsorption isotherm of g-C_3_N_4_/TiO_2_ intercalated composite adsorbent after curve fitting with adsorption models of Langmuir and Freundlich at 310 K. The parameters for Langmuir and Freundlich models are revealed in Table S2. The R^2^ of the two can be compared. The R^2^ of the Freundlich adsorption isotherm is 0.9875, which is closer to 1. Therefore, the fitting result is closer to the Freundlich isotherm model. The main theory of the Langmuir adsorption isotherm is to assume that the full adsorption of the adsorption material is monolayer adsorption. Once the adsorption site is filled in the adsorption process, there will be no further adsorption at the site. The heterogeneous system is described by the Freundlich isotherm theory, and it is assumed that the concentration on the adsorbent surface increases with the increase in adsorbent concentration. The equilibrium concentration fitting results display that the adsorption of BR on g-C_3_N_4_/TiO_2_ intercalated composites is a multi-layer adsorption process. Traditional adsorption material, activated carbon, involves mainly physical adsorption, so g-C_3_N_4_/TiO_2_ intercalation composite has more advantages in the adsorption of BR. The adsorption capacity for BR serves as the most critical performance factor for hemoperfusion adsorbents. Therefore, the performance of our material was compared with those reported in the relevant literature (shown in Table S3) [43,44,50,51,52,53,54,55,56]. The results demonstrated that the g-C_3_N_4_/TiO_2_ intercalation composite exhibited superior adsorption capacity, surpassing that of most previously reported materials.

Through the experiments of adsorption selectivity and biocompatibility of BSA, we further demonstrated the application potential of g-C_3_N_4_/TiO_2_ intercalation composite in the field of BR adsorption. As displayed in Figure 3d, when the BSA concentration reaches 50 g·L^−1^, the clear rate remains more than 96% when albumin coexists. In addition, the clearance efficiency and clearance process of bovine serum albumin by single needle were also studied. The clearance rate at high concentration of bovine serum albumin (50 g·L^−1^) was 0.2%, indicating that g-C_3_N_4_/TiO_2_ intercalation composite will not cause obvious albumin loss, and can effectively inhibit the interference of white protein, so g-C_3_N_4_/TiO_2_ intercalation composite has the potential to be applied as a BR adsorbent in hemoperfusion.

The adsorbent in hemoperfusion is usually in direct contact with blood. During the adsorption process, blood compatibility must be maintained so as not to cause red blood cell rupture. Therefore, in order to prove that the g-C_3_N_4_/TiO_2_ intercalation material can be very effectively used in hemoperfusion devices, and will not cause damage to red blood cells, we conducted a hemolysis rate experiment.

The hemolysis rate is calculated using the formula below [57]:

$$\begin{array}{c} Hemolysis\;rate\;\left(\%\right)\\ =\frac{absorbance\;of\;experimental\;group-absorbance\;of\;negative\;control\;group}{absorbance\;of\;positive\;control\;group-absorbance\;of\;negative\;control\;group} \end{array}$$

**Figure 4:**
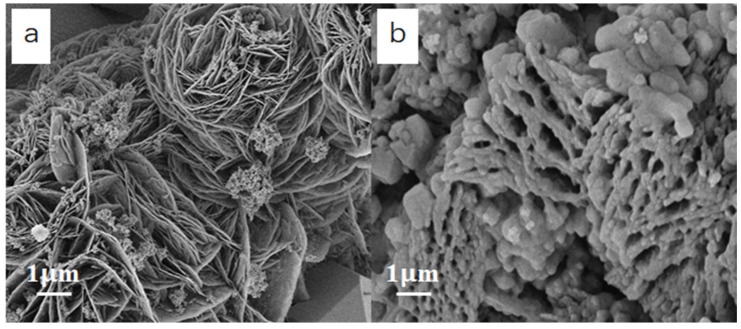
The SEM images of the material before BR adsorption (**a**) and after BR adsorption (**b**).

To further explore the adsorption process of BR by g-C_3_N_4_/TiO_2_ intercalated composites, the morphology of the composites before and after adsorption of BR were observed by SEM. Figure 4 is the SEM comparison diagrams of g-C_3_N_4_/TiO_2_ intercalation composite before and after BR adsorption. Figure 4a displays that the prepared composite presents an expanded carbon nitride lamellar structure before the adsorption process. This structure has a higher specific surface area than the original bulk carbon nitride and can expose more adsorption groups in the middle of the lamella, such as hydroxyl, carboxyl and amino groups, which increases the contact area and probability of protein element functional groups in BR, which is conducive to the adsorption process. Figure 4b is the SEM diagram of the material after BR adsorption. It is clear that a large amount of adsorbed material is BR and that the majority is adsorbed onto the surface of the g-C_3_N_4_/TiO_2_ intercalated composites. The strong adsorption capacity makes BR almost wrap the composites in all directions. Additionally, the base material is composed of g-C_3_N_4_/TiO_2_ intercalated composites, and BR is adsorbed on g-C_3_N_4_/TiO_2_ intercalated composites.

**Figure 5:**
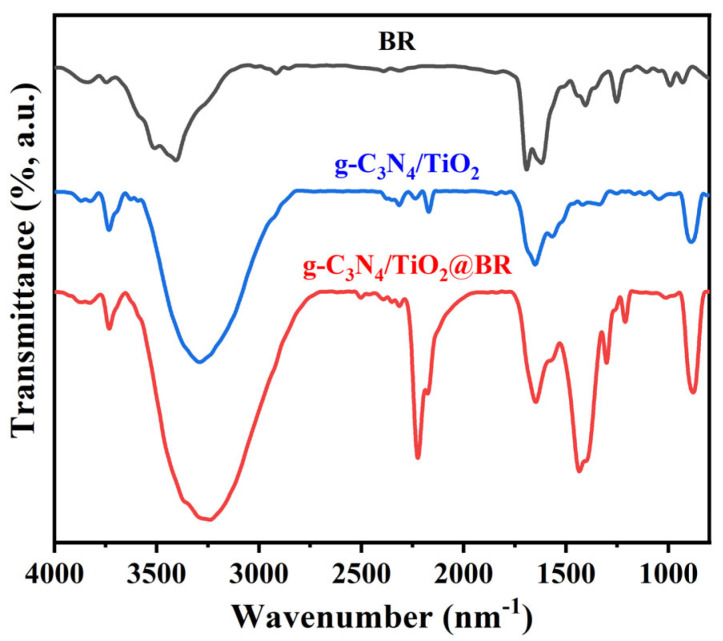
Infrared spectrum of BR and g−C_3_N_4_/TiO_2_ before and after adsorption.

In general, the adsorption of the adsorbed material is mainly owed to the physicochemical properties of the adsorbents, such as pore structure and the combination of surface functional groups. Most of them are physical adsorption as well as van der Waals force. To explore the adsorption mechanism of g-C_3_N_4_/TiO_2_ intercalated composites on BR, we conducted a series of in-depth studies. Figure 5 displays the FT-IR spectra of BR and g-C_3_N_4_/TiO_2_ intercalation composites before and after BR adsorption. It displays that after g-C_3_N_4_/TiO_2_ adsorbs BR, the peak at 1695 cm^−1^ corresponding to N-H bond in BR moves toward to 1651 cm^−1^. The large displacement of the N-H bond displays that the formation of the N-H X-Ti hydrogen bond affects the transfer of the hydrogen electron donor group absorption peak to a lower position [58].

**Figure 6:**
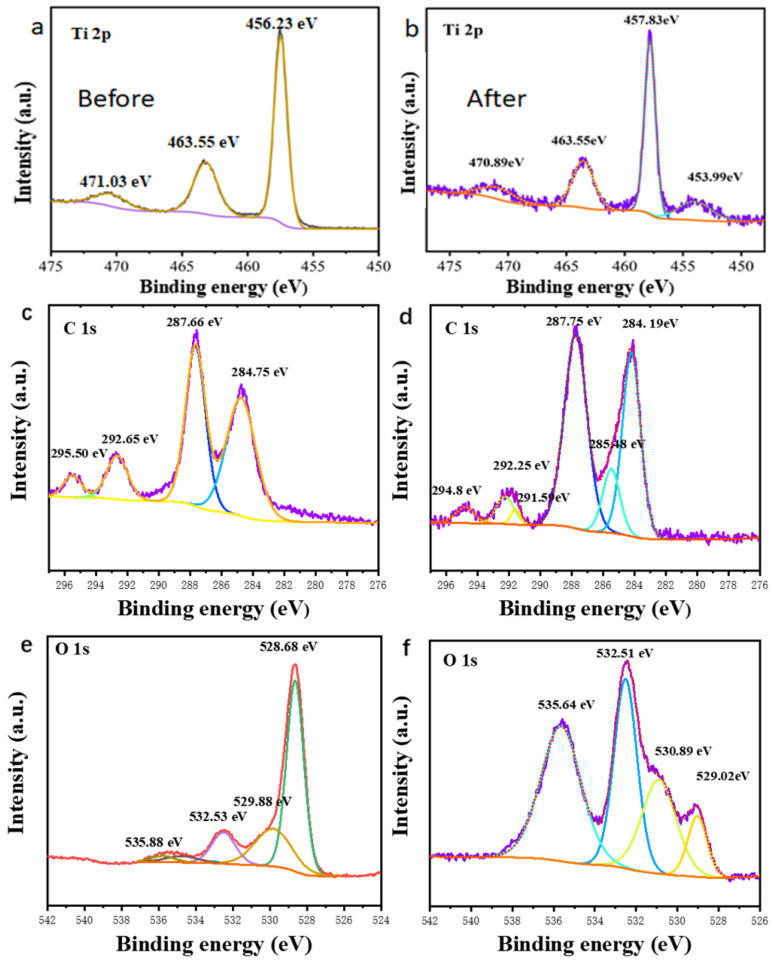
XPS spectra (**a**–**f**) of Ti 2p, C 1s, O 1s before and after the g-C_3_N_4_/TiO_2_ composite material adsorbed BR.

The chemical adsorption details and affinity between g-C_3_N_4_/TiO_2_ intercalated composite adsorbent and BR molecules were researched by XPS. As revealed in Figure 6a,b, the Ti 2p high-resolution spectrum shows the typical binding energy of anatase titanium dioxide before the adsorption behavior occurs, while after the adsorption of BR the binding energy at 471.03 eV decreased by 0.14 eV to about 470.89 eV. The absorption peak at 453.99 eV was due to the absorption peak of Na because the BR solution was prepared by normal saline. Figure 6c,d show the spectra of C 1s before and after adsorption. The peaks at 295.50 eV, 292.65 eV and 284.75 eV decreased to 294.80 eV, 292.25 eV and 284.19 eV, respectively, and decreased by 0.7 eV, 0.4 eV and 0.56 eV, respectively, due to the hydrogen bond formed by BR adsorbed by the adsorbent. In addition, Figure 6e,f show the spectra of O 1s before and after adsorption. The peaks at 535.88 eV and 532.53 eV are shifted downward by 0.24 EV, 0.02 eV to 535.64 eV and 532.51 eV, respectively. This obvious reduction in binding energy is due to a hydrogen bond forming.

The blood perfusion material must possess good blood compatibility for clinical application. To verify whether the g-C_3_N_4_/TiO_2_ composite material induces apoptosis, necrosis, or cytotoxicity in human cells, a cytotoxicity test was conducted using African green monkey kidney cells (Vero cells) and the CCK-8 assay kit. In accordance with the toxicity classification method outlined in the United States Pharmacopeia (USP), cytotoxicity was evaluated based on cell survival rate (Relative Growth Rate, RGR). The evaluation criteria were defined as follows: RGR ≥ 75% indicates compliance. The RGR value was calculated using the following formula:

RGR = (absorbance of sample group/absorbance of blank control group) × 100%.

**Figure 7:**
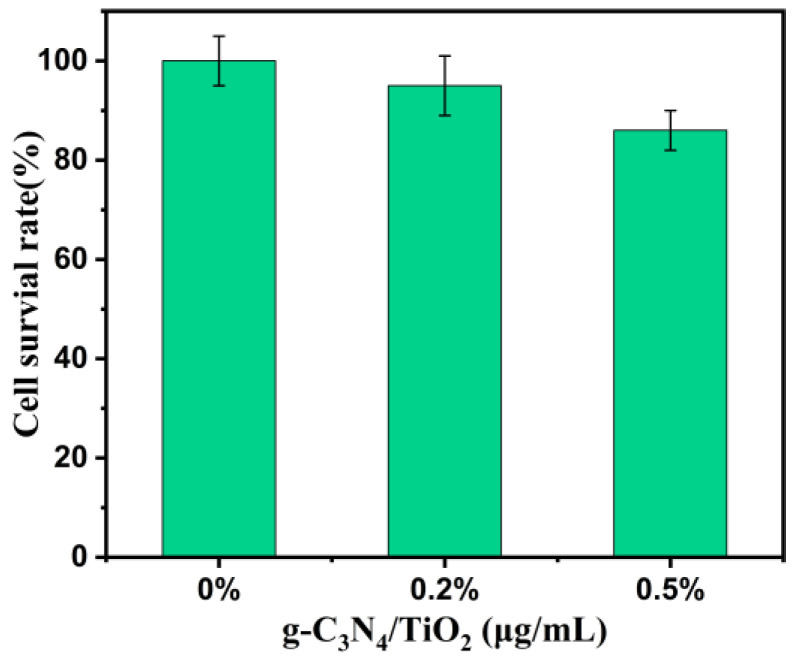
Cytotoxicity test of g-C_3_N_4_/TiO_2_ composite solution.

As shown in Figure 7, the Vero cells were placed in culture medium containing different density g-C_3_N_4_/TiO_2_ intercalation composite material and incubated for 12 h. Then, the degree of cell survival was detected. At the concentration of 0.5 μg·mL^−1^ g-C_3_N_4_/TiO_2_ intercalation composite, the cell survival rate is still above 80%, which proves that the g-C_3_N_4_/TiO_2_ intercalation composite has good biocompatibility.

**Figure 8:**
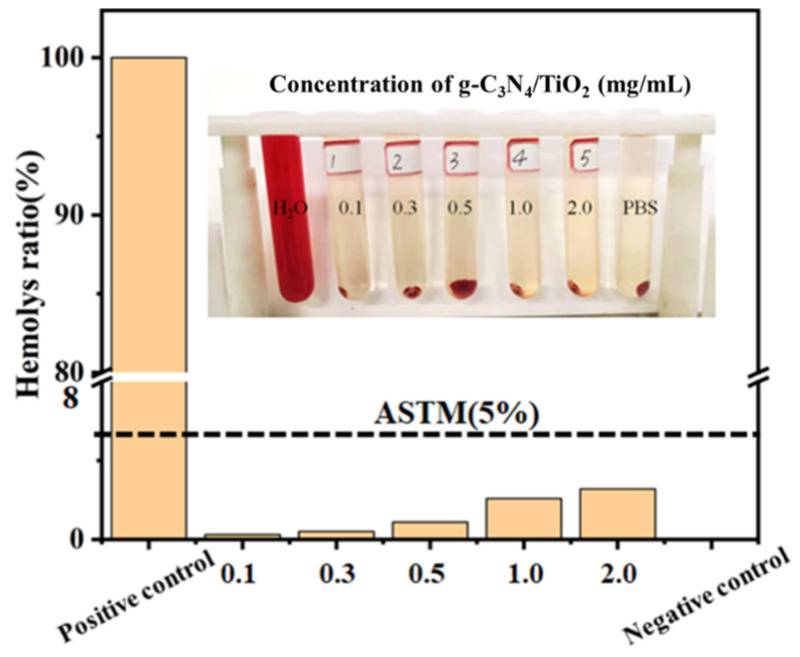
The hemolysis rate results of different samples in the control group and different concentrations of g-C_3_N_4_/TiO_2_ composite materials.

In blood perfusion systems, adsorbents are typically in direct contact with blood. During the adsorption process, it is critical to maintain blood compatibility to prevent adverse effects such as red blood cell rupture (hemolysis). To validate the applicability of the g-C_3_N_4_/TiO_2_ intercalated composite material in blood perfusion devices and confirm its non-damaging effects on erythrocytes, we conducted a hemolysis rate test. As shown in Figure 8, compared with the solution color of negative and positive control groups, no obvious hemolysis was observed even when the concentration of g-C_3_N_4_/TiO_2_ intercalation was 2 mg·mL^−1^. The hemolysis rate of g-C_3_N_4_/TiO_2_ intercalation composite material at the concentration is 1.1%, which is far lower than the standard 5%, which was established by the American Society for Testing and Materials. The above biological experiments show that the g-C_3_N_4_/TiO_2_ intercalation composite material has excellent blood compatibility, has no cytotoxicity and will not cause hemolysis, and can be used as a hemoperfusion material in hemoperfusion.

**Figure 9:**
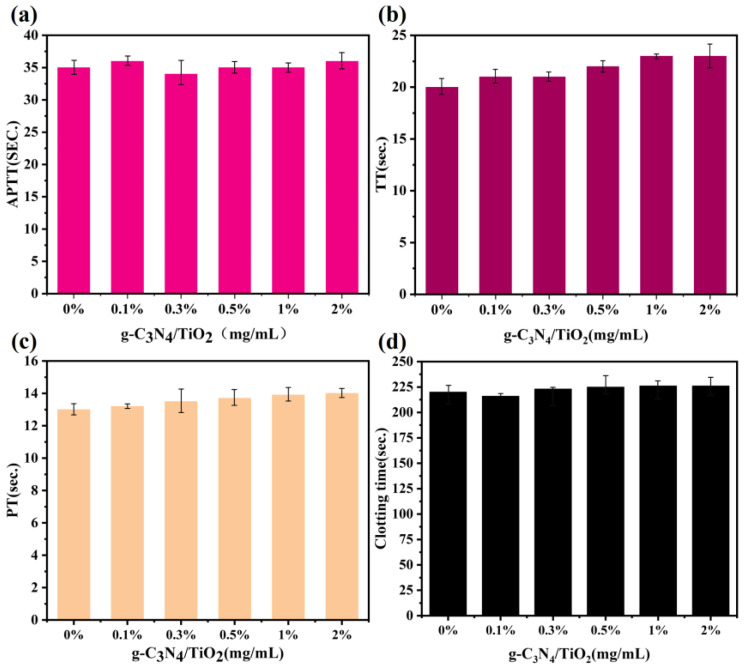
(**a**) APTT, (**b**) TT, (**c**) PT and (**d**) whole blood clotting time values for different amounts of g-C_3_N_4_/TiO_2_ absorbents.

To explore the blood compatibility of the g-C_3_N_4_/TiO_2_ composite and determine whether it affects blood coagulation and platelet activation, coagulation time and platelet factor 4 (PF4) activation experiments were conducted. The coagulation time experiments included activated partial thromboplastin time (APTT), thrombin time (TT), prothrombin time (PT), and whole blood coagulation time, which can reveal the coagulation characteristics of the g-C_3_N_4_/TiO_2_ intercalated composite. In Figure 9, APTT, TT, and PT in blood after pre-incubation with the g-C_3_N_4_/TiO_2_ intercalated composite at different concentrations were detected. APTT and TT can assess the in vitro antithrombotic properties of the sample, while PT reflects exogenous coagulation performance. Compared with the control group, no significant decrease was observed in APTT, TT, or PT values for the g-C_3_N_4_/TiO_2_ composite at different doses (0.1~2 mg∙mL^−1^). The experimental results indicate that the whole blood coagulation time detected after pre-incubation with the g-C_3_N_4_/TiO_2_ intercalated composite at various concentrations showed minimal variation. Compared with the control group, the whole blood coagulation time did not significantly shorten even at a high density of 2 mg∙mL^−1^, indicating that the g-C_3_N_4_/TiO_2_ intercalated composite does not induce significant coagulation.

**Figure 10:**
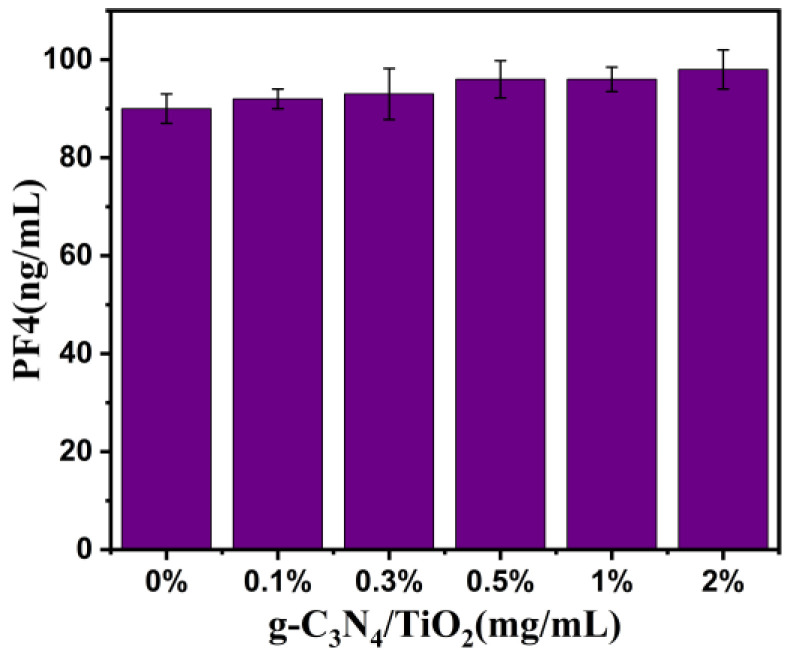
The concentrations of PF4 in whole blood after incubation with different amounts of g-C_3_N_4_/TiO_2_ absorbents.

To investigate whether the g-C_3_N_4_/TiO_2_ composite material induces platelet activation in whole blood, the concentration of platelet factor 4 (PF4) in whole blood pre-incubated with this composite was measured to assess its effect on platelet activation. As revealed in Figure 10, even at a high composite concentration of 2 mg∙mL^−1^, the PF4 concentration exhibited minimal increase compared with the control group. This indicates that the g-C_3_N_4_/TiO_2_ intercalation composite material does not induce platelet activation.

To study the stability of the g-C_3_N_4_/TiO_2_ material under physiologic conditions, the XRDs of the material before and after the adsorption of BR were tested. As shown in Figure S1, the results demonstrate that there is almost no change in the XRD of the material before and after the adsorption for BR. This fully proves the stability of the material under physiological conditions.

## 3. Materials and Methods

### 3.1. g-C_3_N_4_/TiO_2_ Absorbent Preparation

In this work, the g-C_3_N_4_/TiO_2_ was prepared based on the published method and modified partially [59,60]. In the preparation process, melamine was used as the precursor. First, 1.2 g melamine, 4.8 g anatase TiO_2_ and 3.6 g sodium bicarbonate (NaHCO_3_) were added to a beaker, followed by 50 mL of deionized water at 40 °C. Next, the mixed solution was ultrasonically stirred for 60 min. The moisture was then removed by rotary evaporator at 55 °C, yielding a white powder. The powder was ground and placed in an alumina crucible and calcined at 550 °C for 3 h in a tube furnace at a heating rate of 3 °C/min. After cooling to room temperature, the resulting white powder was ground in an agate mortar and washed multiple times with deionized water at 35 °C to remove residual impurities. After this, the final target product was produced.

### 3.2. Adsorption Experiment

In order to investigate the removal of BR by the intercalated composites, the adsorption of intercalated composites in BR solution was measured. According to the relevant literature reports and clinical studies, the total BR level in healthy human serum is generally 2–20 μmol∙L^−1^*;* however, the bodies of patients with liver failure cannot remove the BR in time, so the total BR in patients’ blood samples may rise to 170 and above μmol∙L^−1^, while the conversion unit concentration is about 100 mg∙L^−1^ [61]. Therefore, in order to obtain representative adsorption data, we use 150 mg∙L^−1^ higher than the upper limit of total BR concentration in human blood samples when liver function is damaged as the actual experimental concentration of adsorbed BR. When the patient’s liver function is damaged, the body will have an immune response, and the body temperature will rise slightly. Therefore, we set the temperature of the experiment to 37 °C to simulate the body’s ambient temperature when the human liver function is damaged. In order to simulate the real human body environment, we use the configured phosphoric acid buffer solution to configure the BR solution, so that the pH is close to that of human blood (pH = 7.2~7.4). In order to prevent BR from being oxidized by light, all BR solutions used in the experiment were stored in the dark, wrapped with aluminum foil and placed in a brown glass volumetric flask [51]. BR is easily decomposed under sunlight, so full experiments in BR solution were conducted in a dark condition at 37 °C.

### 3.3. Adsorption Kinetics Experiment

To explore the adsorption kinetics, we took a certain mass of g-C_3_N_4_/TiO_2_ intercalated composite and placed it in a 5 mL centrifuge tube. In the centrifuge tube, we added 3 mL of BR solution with a concentration of 150 mg L^−1^, vibrated evenly, and carried out static adsorption at 37 °C for 1, 5, 15, 30, 60 and 120 min, shaking several times during the adsorption process to make the BR be in uniform contact with the adsorbent [62]. Samples were taken after different adsorption times, and the adsorbent was filtered through a water filtration membrane. The absorbance of the clear solution was measured on a UV–Vis spectrophotometer at a wavelength of 438 nm. The final BR solution concentration was obtained by comparing the absorbance with that in the original solution through the BR standard curve. The adsorption amount of BR by g-C_3_N_4_/TiO_2_ intercalated composite was obtained through the following formula:

(1) $$Q_{e}=\frac{\left(C_{0}-C_{e}\right)\times V}{m}$$

where *c*_0_ represents the initial concentration, *c_e_* represents the equilibrium concentration, V represents the volume of suspension, m represents the mass of adsorbent, *Q_e_* represents the equilibrium adsorption capacity. All experiments were conducted with AC adsorbent as comparison. Full data was the average value of repeated determination or three determinations, and the relative error was about 5%.

### 3.4. Adsorption Isotherm Experiment

The adsorption isotherm describes the functional relevance between the Ce of the solution and the Q of the adsorbent when the adsorption reaches equilibrium. The fitting data results can reflect the maximum adsorption to a certain extent. The adsorption isotherm model also represents the concentration relationship curve between the two-phase interface when the adsorption target reaches equilibrium at a certain temperature.

To make a more particular knowledge of the adsorption rate of BR on g-C_3_N_4_/TiO_2_ intercalated composites, the adsorption results were linearly fitted by quasi first-order and second-order kinetic models. The equations are (2) and (3):

(2) $$log\left(q_{e}-q_{t}\right)=logq_{e}-\frac{K_{1}t}{2.303}$$

(3) $$\frac{t}{q_{t}}=\frac{1}{k_{2}q_{e}^{2}}+\frac{t}{q_{e}}$$

where $q_{e}$ represent the equilibrium adsorption capacity at time t, $q_{t}$ represents the adsorption capacity at time t, *K*_1_ and *K*_2_ are the rate constants of the quasi first-order and quasi second-order adsorption kinetic equations, respectively.

To further study whether the adsorption of BR by the adsorbent prepared by us is chemical adsorption or physical adsorption, we established representative surface adsorption models of single solute system, the Langmuir adsorption isotherm model and the Freundlich adsorption isotherm model. In detail, the Langmuir model usually assumes that there is no interaction between the adsorbed material and the adsorbed material molecules, and the adsorption between them belongs to monolayer molecular adsorption. However, the Freundlich model is an empirical model, which is based on heterogeneous surface adsorption. The Freundlich isotherm model considers several adsorption positions on solids and appropriately represents the adsorption data at low and medium concentrations on heterogeneous surfaces. The higher correlation coefficient (R^2^) indicates that the applicability of the isotherm model is higher. The two adsorption models are shown as follows:

(4) $$\frac{c_{e}}{q_{e}}=\frac{c_{e}}{q_{max}}+\frac{1}{K_{L}q_{max}}$$

(5) $$lnqe=\frac{1}{n}lnc_{e}+lnK_{F}$$

where *c_e_* represents the concentration when the ion was in equilibrium, *q_e_* represents the equilibrium adsorption capacity, *q_max_* represents the maximum adsorption capacity of the whole monolayer in the Langmuir model, *K_L_* represents the Langmuir constant, and *K_F_* represents the Freundlich constant.

### 3.5. Competitive Adsorption Experiment

To investigate competitive adsorption, we conducted the following experiment: A measured mass of g-C_3_N_4_/TiO_2_ intercalated composite material was placed into a centrifuge tube (5 mL). Each tube was added with 3 mL of BR solution (initial concentration: 150 mg∙L^−1^) and varying concentrations of BSA. The mixtures were allowed to adsorb under static conditions in a dark room at 37 °C for 2 h. During adsorption, the tubes were shaken intermittently to ensure uniform contact between BR, BSA, and the adsorbent. After reaching adsorption equilibrium, samples were collected. The adsorbent was separated by filtration through an aqueous filter membrane of 0.45 μm. Then, the clear filtrate was analyzed using a UV-Vis spectrophotometer at 438 nm. BR and BSA concentrations were determined by comparing absorbance values with their respective standard curves and the original solution’s absorbance. Finally, the adsorption capacity of the g-C_3_N_4_/TiO_2_ for bilirubin and BSA was calculated using the predefined formula based on concentration changes.

### 3.6. Cell Toxicity Experiment

The hemoperfusion material should be used in-clinic, so the hemoperfusion material must have good blood compatibility. In order to verify whether the g-C_3_N_4_/TiO_2_ intercalation composite material can be effectively used in hemoperfusion, we tested whether it will cause the apoptosis and necrosis of human cells and toxicity to the cells. The cells we used in the cytotoxicity experiment were the African green ape kidney cells (Vero cells). Additionally, the cck-8 kit (Sigma-Aldrich, St. Louis, MI, USA) was used in the cytotoxicity experiment of g-C_3_N_4_/TiO_2_ intercalation composite material. We referred to the “United States Pharmacopoeia” toxicity classification method according to the cell survival rate, evaluated the cytotoxicity, and the evaluation standard is as follows: the cell survival rate ≥ 75% qualified.

The calculation formula of cell survival rate is as follows [63]:

$$Cell\;survival\;rate=\frac{absorbance\;value\;of\;sample\;group}{absorbance\;value\;of\;blank\;control\;group}\times 100\%$$

## 4. Conclusions

In summary, g-C_3_N_4_ and TiO_2_ nanoparticles were combined as hemoperfusion adsorbents to adsorb BR toxins for the first time. The composite was compared and evaluated with the market-oriented hemoperfusion adsorbent and activated carbon as well. The theoretical maximum adsorption capacity Q of the composite is as high as 850 mg·g^−1^. The removal effect, the adsorption kinetics and adsorption isotherm were explored. It was found that the adsorption accorded with the quasi second-order kinetics, indicating that is the chemical adsorption process. Then, XPS results prove that there is a strong chemical interaction between g-C_3_N_4_/TiO_2_ intercalation composite and BR, which is mainly reflected in the formation of hydrogen bonds between the adsorbent and target after adsorption. Furthermore, the adsorption process conforms to Freundlich isotherm theory. It demonstrates this adsorption is a multi-layer adsorption process. The g-C_3_N_4_/TiO_2_ intercalated composite has more advantages than activated carbon and holds significant promise for applications in hemoperfusion.

## Data Availability

All data are available in the main text or the Supplementary Materials.

## Supplementary Materials

The following supporting information can be downloaded at: https://www.mdpi.com/article/10.3390/molecules30132729/s1, Table S1. The detailed pore structure parameters of TiO_2_, g-C_3_N_4_ and g-C_3_N_4_/TiO_2_ composite.; Table S2. Parameters for Langmuir and Freundlich models of adsorption on g-C_3_N_4_/TiO_2_ composite.; Table S3. Comparison of bilirubin adsorption capacities with other reported adsorbents.; Figure S1. XRD of the g-C_3_N_4_/TiO_2_ material before and after the adsorption of BR. References [43,44,50,51,52,53,54,55,56] are cited in Supplementary Materials.

## References

[1] Kwo P.Y., Cohen S.M., Lim J.K. (2017). ACG Clinical Guideline: Evaluation of Abnormal Liver Chemistries. Am. J. Gastroenterol..

[2] Mironczuk I., Witkowska A.M., Zujko M.E. (2018). Endogenous non-enzymatic antioxidants in the human body. Adv. Med. Sci..

[3] Toyoda H., Johnson P.J. (2022). The ALBI score: From liver function in patients with HCC to a general measure of liver function. Jhep Rep..

[4] Hall B., Levy S., Dufault-Thompson K., Arp G., Zhong A., Ndjite G.M., Weiss A., Braccia D., Jenkins C., Grant M.R. (2024). BilR is a gut microbial enzyme that reduces bilirubin to urobilinogen. Nat. Microbiol..

[5] Wu G., Zhang J., Zhao Q., Zhuang W., Ding J., Zhang C., Gao H., Pang D.-W., Pu K., Xie H.-Y. (2020). Molecularly Engineered Macrophage-Derived Exosomes with Inflammation Tropism and Intrinsic Heme Biosynthesis for Atherosclerosis Treatment. Angew. Chem. Int. Edit..

[6] Zhou Z., Guo K., Luo Y., Yang Q., Wu H., Zeng R., Jiang R., Li J., Wei R., Lian Q. (2024). Targeted modulation of intestinal epithelial regeneration and immune response in ulcerative colitis using dual-targeting bilirubin nanoparticles. Theranostics.

[7] Dossou S., Koshio S., Ishikawa M., Yokoyama S., Dawood M.A.O., El Basuini M.F., El-Hais A.M., Olivier A. (2018). Effect of partial replacement of fish meal by fermented rapeseed meal on growth, immune response and oxidative condition of red sea bream juvenile, *Pagrus major*. Aquaculture.

[8] Stocker R., Yamamoto Y., McDonagh A.F., Glazer A.N., Ames B.N. (1987). Bilirubin is an antioxidant of possible physiological importance. Science.

[9] Patel J.J., Taneja A., Niccum D., Kumar G., Jacobs E., Nanchal R. (2015). The association of serum bilirubin levels on the outcomes of severe sepsis. J. Intensive Care Med..

[10] Wlodzimirow K.A., Eslami S., Abu-Hanna A., Nieuwoudt M., Chamuleau R.A.F.M. (2013). A systematic review on prognostic indicators of acute on chronic liver failure and their predictive value for mortality. Liver Int..

[11] Huang S., Zheng J., Zhang Y., Zheng J., Zhuang Z., Yang Q., Wang F., Chen G., Huang S., Ouyang G. (2020). Polydopamine decorated ordered mesoporous carbon for efficient removal of bilirubin under albumin-rich conditions. J. Mater. Chem. B.

[12] He X.M., Carter D.C. (1992). Atomic structure and chemistry of human serum albumin. Nature.

[13] Du Y., Chen M., Wang B., Chai Y., Wang L., Li N., Zhang Y., Liu Z., Guo C., Jiang X. (2024). TiO_2_/Polystyrene nanocomposite antibacterial material as a hemoperfusion adsorbent for efficient bilirubin removal and prevention of bacterial infection. ACS Biomater. Sci. Eng..

[14] Li Q., Zhu Y., Li Y., Yang J., Bao Z., Tian S., Wang X., Zhang L. (2023). Reusable zwitterionic porous organic polymers for bilirubin removal in serum. ACS Appl. Mater. Interfaces.

[15] Liu Y., Wang Z.-K., Liu C.-Z., Liu Y.-Y., Li Q., Wang H., Cui F., Zhang D.-W., Li Z.-T. (2022). Supramolecular organic frameworks as adsorbents for efficient removal of excess bilirubin in hemoperfusion. ACS Appl. Mater. Interfaces.

[16] Wu S., Yue P., Ma Y., Zou Y., Liang W., Ye Q. (2023). Hemoperfusion adsorbents for removal of common toxins in liver and kidney failure: Recent progress, challenges, and prospects. Adv. Mater..

[17] Zhou W., Hu W., Zhan Q., Zhang M., Liu X., Hussain W., Yu H., Wang S., Zhou L. (2023). Novel hemoperfusion adsorbents based on collagen for efficient bilirubin removal—A thought from yellow skin of patients with hyperbilirubinemia. Int. J. Biol. Macromol..

[18] Chen Y., Zhao P., Fan W., Niu J. (2022). Relationship between serum indirect bilirubin levels and cardiovascular events and all-cause mortality in maintenance hemodialysis patients. Ther. Clin. Risk Manag..

[19] Maheshwari V., de Ferris M.E.D.-G., Filler G., Kotanko P. (2024). Novel extracorporeal treatment for severe neonatal jaundice: A mathematical modelling study of allo-hemodialysis. Sci. Rep..

[20] Su H.-H., Kao C.-M., Lin Y.-C., Lin Y.-C., Kao C.-C., Chen H.-H., Hsu C.-C., Chen K.-C., Peng C.-C., Wu M.-S. (2017). Relationship between serum total bilirubin levels and mortality in uremia patients undergoing long-term hemodialysis: A nationwide cohort study. Atherosclerosis.

[21] Zoccali C., Mallamaci F., Adamczak M., de Oliveira R.B., Massy Z.A., Sarafidis P., Agarwal R., Mark P.B., Kotanko P., Ferro C.J. (2023). Cardiovascular complications in chronic kidney disease: A review from the European Renal and Cardiovascular Medicine Working Group of the European Renal Association. Cardiovasc. Res..

[22] Zhang M.J., Liu X.J., Zhou W., Zheng X.L., Wang S.Q., Zhou L. (2023). Ordered porous materials for blood purification. Sep. Purif. Technol..

[23] Zhang L.Q., Liu G.H., Xia Q.P., Deng L. (2024). Research progress on blood compatibility of hemoperfusion adsorbent materials. Front. Bioeng. Biotechnol..

[24] Dou W.Y., Wang J., Yao Z.K., Xiao W., Huang M., Zhang L. (2022). A critical review of hemoperfusion adsorbents: Materials, functionalization and matrix structure selection. Mater. Adv..

[25] Guo C., Jiang X.B., Guo X.F., Ou L.L. (2024). An Evolutionary Review of Hemoperfusion Adsorbents: Materials, Preparation, Functionalization, and Outlook. ACS Biomater. Sci. Eng..

[26] Bakry A.M., Abbas S., Ali B., Majeed H., Abouelwafa M.Y., Mousa A., Liang L. (2016). Microencapsulation of Oils: A Comprehensive Review of Benefits, Techniques, and Applications. Compr. Rev. Food Sci. Food Saf..

[27] Weng K., Xu X., Chen Y., Li X., Qing C., Zou D. (2024). Development and applications of multifunctional microencapsulated PCMs: A comprehensive review. Nano Energy.

[28] Xiang J., Mlambo R., Shaw I., Seid Y., Shah H., He Y., Kpegah J.K.S.K., Tan S., Zhou W., He B. (2023). Cryopreservation of bioflavonoid-rich plant sources and bioflavonoid-microcapsules: Emerging technologies for preserving bioactivity and enhancing nutraceutical applications. Front. Nutr..

[29] Xie A., Zhao S., Liu Z., Yue X., Shao J., Li M., Li Z. (2023). Polysaccharides, proteins, and their complex as microencapsulation carriers for delivery of probiotics: A review on carrier types and encapsulation techniques. Int. J. Biol. Macromol..

[30] Chen S., Bao J., Hu Z., Liu X., Cheng S., Zhao W., Zhao C. (2024). Porous Microspheres as Pathogen traps for sepsis therapy: Capturing active pathogens and alleviating inflammatory reactions. ACS Appl. Mater. Interfaces.

[31] Ke J., Wei Y., Chen B. (2024). Application of Hemoperfusion in the Treatment of Acute Poisoning. Blood Purif..

[32] Li Y., Han M., Yang M., Su B. (2024). Hemoperfusion with the HA330/HA380 Cartridge in Intensive Care Settings: A State-Of-The-Art Review. Blood Purif..

[33] Wang L., Han K., Jiang X., Mao C., Li X., Zhou M. (2024). Recent advances in blood toxin removal technology. Sustain. Mater. Technol..

[34] Kuryata O., Akimov O., Riabushko M., Kostenko H., Kostenko V., Mishchenko A., Nazarenko S., Solovyova N., Kostenko V. (2024). Therapeutic potential of 5-aminolevulinic acid in metabolic disorders: Current insights and future directions. Iscience.

[35] Zhang J., Zhong Y.K., Zhou Y.P., Zhang Q.Y., Wang S.Y. (2021). Influence of the acid groups on activated carbon on the adsorption of bilirubin. Mater. Chem. Phys..

[36] Fu X.Y., Shi M.S., Chen D.Y., Zhao X.Y., Jiang T.T., Zhao R. (2024). Theory-driven tailoring of the microenvironment of quaternary ammonium binding sites on electrospun nanofibers for efficient bilirubin removal in hemoperfusion. Polymers.

[37] Du Y., Li X., Lv X., Jia Q. (2017). Highly Sensitive and selective sensing of free bilirubin using metal-organic frameworks-based energy transfer process. ACS Appl. Mater. Interfaces.

[38] Lu Z., Xiao Z., Wang J., Zhao Z., Huang J., Cui J., Mei Y., Huang G. (2024). Bimetallic mof-based fibrous device for noninvasive bilirubin sensing. Adv. Mater. Technol..

[39] Nandi S., Biswas S. (2019). A recyclable post-synthetically modified Al(iii) based metal-organic framework for fast and selective fluorogenic recognition of bilirubin in human biofluids. Dalton Trans..

[40] Yang J., Yuan Y., Hou J., Xu Z., Du Y., Yang J., Shan S., Hu T., Wang C., Su H. (2025). Cyclodextrin metal-organic framework derived nano-cubic gel particles for the efficient removal of bilirubin. Sep. Purif. Technol..

[41] Deng S., Nie Y., Du Z., Huang Q., Meng P., Wang B., Huang J., Yu G. (2015). Enhanced adsorption of perfluorooctane sulfonate and perfluorooctanoate by bamboo-derived granular activated carbon. J. Hazard. Mater..

[42] Gayathiri M., Pulingam T., Lee K.T., Sudesh K. (2022). Activated carbon from biomass waste precursors: Factors affecting production and adsorption mechanism. Chemosphere.

[43] Ando K., Shinke K., Yamada S., Koyama T., Takai T., Nakaji S., Ogino T. (2009). Fabrication of carbon nanotube sheets and their bilirubin adsorption capacity. Colloids Surf. B-Biointerfaces.

[44] Shinke K., Ando K., Koyama T., Takai T., Nakaji S., Ogino T. (2010). Properties of various carbon nanomaterial surfaces in bilirubin adsorption. Colloids Surf. B-Biointerfaces.

[45] Ma C.-F., Gao Q., Xia K.-S., Huang Z.-Y., Han B., Zhou C.-G. (2017). Three-dimensionally porous graphene: A high-performance adsorbent for removal of albumin-bonded bilirubin. Colloids Surf. B-Biointerfaces.

[46] Ferreira V.R.A., Azenha M.A., Pereira C.M., Silva A.F. (2022). Molecularly imprinted methyl-modified hollow TiO_2_ microspheres. Molecules.

[47] Yang Z., Zhang C. (2009). Adsorption and photocatalytic degradation of bilirubin on hydroxyapatite coatings with nanostructural surface. J. Mol. Catal. A Chem..

[48] Cai H., Zhang X.-C., Zhang L., Luo C., Lin H.-J., Han D.-M., Chen F.-Z., Huang C. (2024). Molecule engineering metal-organic framework-based organic photoelectrochemical transistor sensor for ultrasensitive bilirubin detection. Anal. Chem..

[49] Lipinska W., Saska V., Siuzdak K., Karczewski J., Zaleski K., Coy E., de Poulpiquet A., Mazurenko I., Lojou E. (2024). Interaction between bilirubin oxidase and Au nanoparticles distributed over dimpled titanium foil towards oxygen reduction reaction. Electrochim. Acta.

[50] Anasori B., Gogotsi Y. (2017). 2D metal carbides and nitrides (Mxene) for energy storage. Nat. Rev. Mater..

[51] Xie M., Sun J., Chen L. (2019). Procion Blue H-5R functionalized cellulose membrane with specific removal of bilirubin. Cellulose.

[52] Xu X., Zhuang F., Wang W., Li N., Zhang X., Wang H., Shi L. (2017). Development of amino acid-modified PET/PA6 segmented pie bicomponent spunbonded microfiber nonwoven for bilirubin affinity adsorption. Fibers Polym..

[53] Li Q., Guo H., Yang J., Zhao W., Zhu Y., Sui X., Xu T., Zhang J., Zhang L. (2020). MOF-based anti-biofouling hemoadsorbent for highly efficient removal of protein-bound bilirubin. Langmuir.

[54] Li Z., Huang X., Wu K., Jiao Y., Zhou C. (2020). Fabrication of regular macro-mesoporous reduced graphene aerogel beads with ultra-high mechanical property for efficient bilirubin adsorption. Mater. Sci. Eng. C.

[55] Li Q., Zhao W., Guo H., Yang J., Zhang J., Liu M., Xu T., Chen Y., Zhang L. (2020). Metal-organic framework traps with record-high bilirubin removal capacity for hemoperfusion therapy. ACS Appl. Mater. Interfaces.

[56] Zhao R., Ma T., Cui F., Tian Y., Zhu G. (2020). Porous aromatic framework with tailored binding sites and pore sizes as a high-performance hemoperfusion adsorbent for bilirubin removal. Adv. Sci..

[57] Wang T., Sun X., Guo X., Zhang J., Yang J., Tao S., Guan J., Zhou L., Han J., Wang C. (2021). Ultraefficiently calming cytokine storm using Ti_3_C_2_T x MXene. Small Methods.

[58] Song X., Xu T., Yang L., Li Y., Yang Y., Jin L., Zhang J., Zhong R., Sun S., Zhao W. (2020). Self-anticoagulant nanocomposite spheres for the removal of bilirubin from whole blood: A step toward a wearable artificial liver. Biomacromolecules.

[59] Algara-Siller G., Severin N., Chong S.Y., Bjorkman T., Palgrave R.G., Laybourn A., Antonietti M., Khimyak Y.Z., Krasheninnikov A.V., Rabe J.P. (2014). Triazine-based graphitic carbon nitride: A two-dimensional semiconductor. Angew. Chem. Int. Edit..

[60] Tao S.X., Wang T.Y., Wu Y.H., Wang C.Y., Wang G.X. (2021). Removal of extremely low concentration cobalt by intercalation composite material of carbon nitride/titanium dioxide. J. Hazard. Mater.

[61] Wei H., Han L., Tang Y., Ren J., Zhao Z., Jia L. (2015). Highly flexible heparin-modified chitosan/graphene oxide hybrid hydrogel as a super bilirubin adsorbent with excellent hemocompatibility. J. Mater. Chem. B.

[62] Wu K., Liu X., Li Z., Jiao Y., Zhou C. (2020). Fabrication of chitosan/graphene oxide composite aerogel microspheres with high bilirubin removal performance. Mater. Sci. Eng. C Mater. Biol. Appl..

[63] Esteves-Pedro N.M., Sugibayashi K., Ostrosky E.A., Ferrari M., Sufi B.d.S., Mathor M.B., Moreno P.R.H., Lourenco F.R., Consiglieri V.O., Baby A.R. (2018). Validation cytotoxicity assay for lipophilic substances. Curr. Top. Med. Chem..

