# Supplementary material for: Heterojunction-Engineered g-C3N4/TiO2 Nanocomposites with Superior Bilirubin Removal Efficiency for Enhanced Hemoperfusion Therapy
Source: Molecules. 2025 Jun 25;30(13):2729. doi: 10.3390/molecules30132729 (PMC12251008; doi:10.3390/molecules30132729)
Supplement: Supplementary file 1 [file molecules-30-02729-s001.zip › molecules-3610704-supplementary.docx]

**Supplementary Materials**

**Heterojunction-Engineered g-C_3_N_4_/TiO_2_ Nanocomposites with Superior Bilirubin Removal Efficiency for Enhanced Hemoperfusion Therapy**

Lingdong Meng ^1†^, Shouxuan Tao ^2†^, Liyao Wang ^3^, Yu Cao ^4*^, Jianhua Hou ^4*^ and Chengyin Wang ^2*^

^1^ Hemodialysis Center of Yangzhou Hospital of Traditional Chinese Medicine, Yangzhou 225000, China; mengling-dong1982@163.com

^2^ College of Chemistry and Chemical Engineering, Yangzhou University, Yangzhou 225000, China; shouxuantao@foxmail.com

^3^ Faculty of Materials Science and Chemistry, China University of Geosciences, Wuhan 430074, China; WangLiYao@cug.edu.cn

^4^ College of Environmental Science and Engineering, Yangzhou University, Yangzhou 225000, China;

**^*^** Correspondence: yucao@yzu.edu.cn (Y.C.); jhhou@yzu.edu.cn (J.H.); wangcy@yzu.edu.cn (C.W.)

^†^ These authors contributed equally to this work.

**Materials**

Melamine (C_3_H_6_N_6_), anatase titanium dioxide, sodium bicarbonate (NaHCO_3_), bilirubin (BR), sodium hydroxide (NaOH) and bovine serum albumin (BSA) were purchased from Maclin Biochemical Technology Co., LTD (Shanghai, China). No further purification procedures are carried out, all materials are utilized directly in their received condition.

**Characterization**

X-ray diffraction (XRD) patterns of the materials were recorded using a Rigaku D/max-2550 diffractometer with Cu Kα radiation (λ = 1.5418 Å) at 50 KV and 200 mA. The scattering signatures were obtained at 2θ intervals of 0.02° and at a scan speed of 2° per minute. The morphologies of the obtained products were recorded on a scanning electron microscopy (SEM) by using Supra 55VP (Zeiss, Germany). N_2_ adsorption-desorption isotherms were measured at 77 K by using an ASAP 2460 analyzer (Micromeritics, USA). The Brunauer-Emmett-Teller (BET) surface area was calculated using adsorption data in a relative pressure ranging from 0.05 to 0.30. The pore size distributions were estimated by using NLDFT method. TEM images of the samples were obtained on a JEM-2100 transmission electron microscope (JEOL, Japan). The elemental distributions of the materials were characterized by Transmission Electron Microscopy-Energy Dispersive X-ray Spectroscopy (TEM-EDX, FEI Tecnai G20 F30 S-TWIN, USA). Fourier Transform infrared spectrum (FT-IR): The above-mentioned freeze-dried g-C_3_N_4_/TiO_2_ adsorbed BR sample was ground in KBr powder, and then characterized by FT-IR (Tensor 27, Germany). The X-ray photoelectron spectroscopy (XPS) spectra were recorded on an ESCALAB 250 instrument (Thermo-VG Scientific, USA) equipped with a monochromatized Al Kα excitation source (hν = 1486.6 eV). The C 1s peak (284.6 eV) was used for the calibration of binding energy values.

**Table S1.** The detailed pore structure parameters of TiO_2_, g-C_3_N_4_ and g-C_3_N_4_/TiO_2_ composite.

| **Materials** | **Surface area**  **(m^2^∙g^-1^)** | **Pore volume**  **(cm^3^∙g^-1^)** | **Pore diameter**  **(nm)** |
| --- | --- | --- | --- |
| TiO_2_  g-C_3_N_4_ | 24.37  7.68 | 0.111  0.039 | 4.178  3.709 |
| g-C_3_N_4_/TiO_2_ | 34.52 | 0.145 | 3.138 |

**Table S2.** Parameters for Langmuir and Freundlich models of adsorption on g-C_3_N_4_/TiO_2_ composite.

| T(K) | Langmuir constants | |  | Freundlich constants | |  |
| --- | --- | --- | --- | --- | --- | --- |
|  | *K*_L_ | *q*_max_ | R^2^ | n | *K*_F_ | R^2^ |
| 310 | 0.089 | 1199.47 | 0.9834 | 0.327 | 21.338 | 0.9875 |

**Table S3**. Comparison of bilirubin adsorption capacities with other reported adsorbents.

| Adsorption material | Adsorption capacity (mg/g) | Ref. |
| --- | --- | --- |
| Activated carbon | 23 | [1] |
| Carbon nanotube-chitosan composite beads | 43.6 | [2] |
| chitosan/graphene oxide  composite aerogel microspheres | 92.59 | [3] |
| Procion Blue H-5R functionalized cellulose membrane | 230 | [4] |
| PET/PA6 SBSNW-Lys | 388.69 | [5] |
| MOF-based  anti-biofouling hemoadsorbent | 583 | [6] |
| Macro-mesoporous reduced graphene aerogel beads | 649.52 | [7] |
| PCN-333 | 1003.8 | [8] |
| Porous aromatic framework | 1249 | [9] |
| g-C_3_N_4_/TiO_2_ intercalation composite | 1199.47 | **This work** |

**References**

1. Ando, K.; Shinke, K.; Yamada, S.; Koyama, T.; Takai, T.; Nakaji, S.; Ogino, T. Fabrication of carbon nanotube sheets and their bilirubin adsorption capacity. *Colloids Surf. B-Biointerfaces* **2009**, *71*, 255-259.

2. Shinke, K.; Ando, K.; Koyama, T.; Takai, T.; Nakaji, S.; Ogino, T. Properties of various carbon nanomaterial surfaces in bilirubin adsorption. *Colloids Surf. B-Biointerfaces* **2010**, *77*, 18-21.

3. Anasori, B.; Gogotsi, Y. 2D metal carbides and nitrides (Mxene) for energy storage. *Nat. Rev. Mater.* **2017**, 2, 1-17.

4. Xie, M.; Sun, J.; Chen, L. Procion Blue H-5R functionalized cellulose membrane with specific removal of bilirubin. *Cellulose* **2019**, 26, 8073-8085.

5. Xu, X.; Zhuang, F.; Wang, W.; Li, N.; Zhang, X.; Wang, H.; Shi, L. Development of amino acid-modified PET/PA6 segmented pie bicomponent spunbonded microfiber nonwoven for bilirubin affinity adsorption. *Fibers Polym*. **2017**, 18, 633-640.

6. Li, Q.; Guo, H.; Yang, J.; Zhao, W.; Zhu, Y.; Sui, X.; Xu, T.; Zhang, J.; Zhang, L. MOF-based anti-biofouling hemoadsorbent for highly efficient removal of protein-bound bilirubin. *Langmuir* **2020**, 36,‏ 8753-8763.

7. Li, Z.; Huang, X.; Wu, K.; Jiao, Y.; Zhou, C. Fabrication of regular macro-mesoporous reduced graphene aerogel beads with ultra-high mechanical property for efficient bilirubin adsorption. *Materials Science & Engineering C*, **2020**, 106, 110282-110293.

8. Li, Q.; Zhao, W.; Guo, H., Yang, J.; Zhang, J.; Liu, M.; Xu, T.; Chen, Y.; Zhang, L. Metal-organic framework traps with record-high bilirubin removal capacity for hemoperfusion therapy. *ACS Appl. Mater. Interfaces* **2020**, 12, 25546-25556.

9. Zhao, R.; Ma, T.; Cui, F.; Tian, Y.; Zhu, G. Porous aromatic framework with tailored binding sites and pore sizes as a high-performance hemoperfusion adsorbent for bilirubin removal. *Adv. Sci*. **2020**, 7, 2001899-2001907.


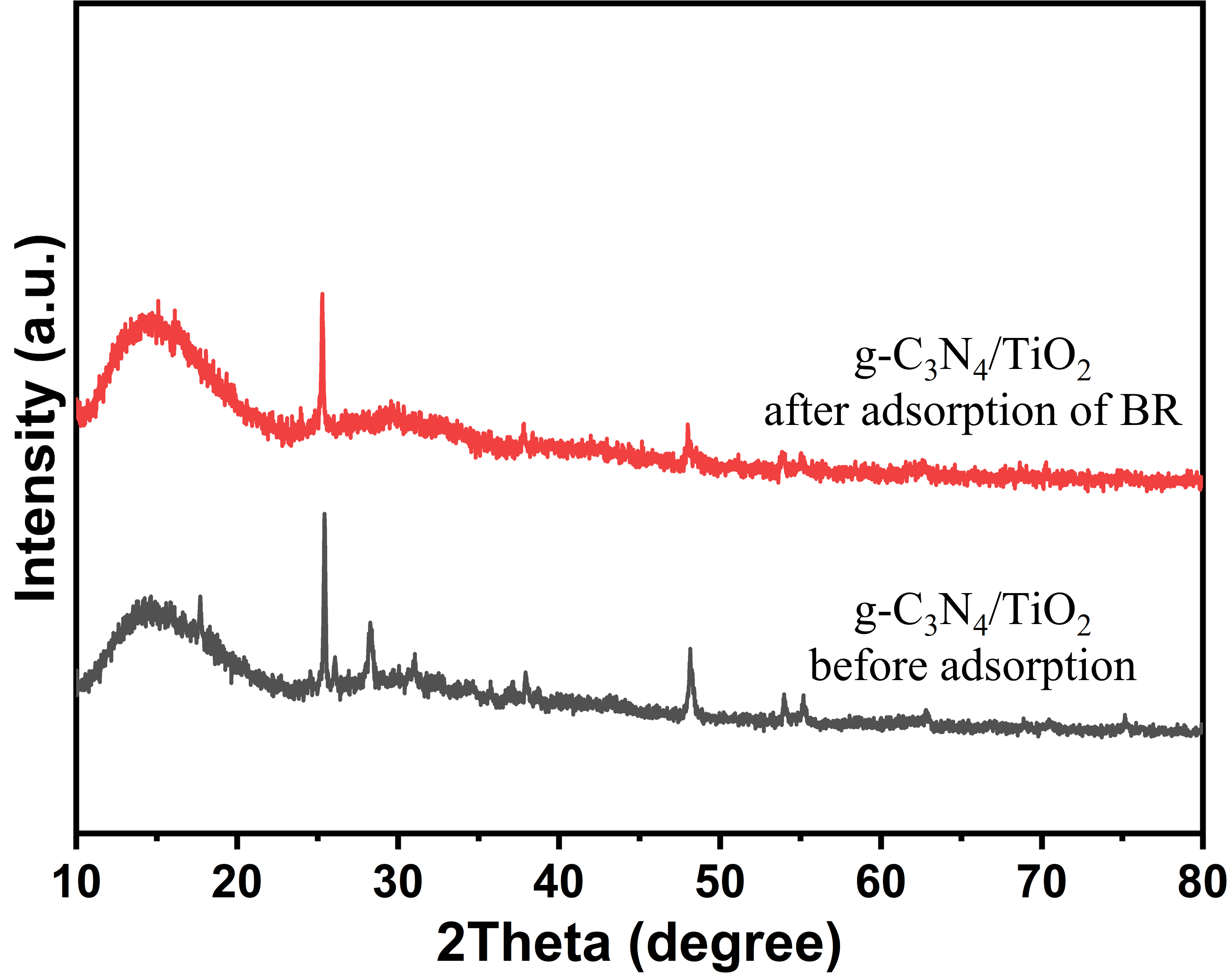


**Figure S1.** XRD of the g-C_3_N_4_/TiO_2_ material before and after the adsorption of BR.
